# Diabetes: What Challenges Lie Ahead for Vietnam?

**DOI:** 10.5334/aogh.2526

**Published:** 2020-01-02

**Authors:** Nguyen Bich Ngoc, Zhou Lu Lin, Waqas Ahmed

**Affiliations:** 1 Jiangsu University, School of Management, Zhenjiang, CN; 2 Westminster International University in Tashkent, UZ

## Abstract

**Background:**

Economic development and social environment changes influence disease patterns ranging from infectious diseases to noncommunicable diseases, and diabetes is one of the seven causes leading to death and disability in Vietnam.

**Objectives:**

The purpose of this research is to present an overview of the challenges related to diabetes prevention in Vietnam and to find effective ways for the prevention and control of diabetes, as well as to improve the quality of life among diabetes patients.

**Methods:**

The literature review was conducted using a variety of databases, such as PubMed, Google Scholar, Science Direct, Vietnamese data sources, and papers published in the Vietnamese language. For the searches, we used keywords such as “Diabetes,” “Prevention,” and “Prevalence of Diabetes.”

**Findings and Conclusions:**

With the increasing prevalence of diabetes, there are approximately 5.76 million people with diabetes currently living in Vietnam. The age-adjusted comparative prevalence of diabetes in the population of Vietnam was approximately 6% in 2017. This review suggests that the government needs to establish social security and policy programs aimed at reducing social risk factors and the burden of healthcare costs for diabetes treatment in older people. In addition, attention should be paid to the management and control of diabetes-related diseases, with an emphasis on new techniques for early diagnosis and treatment. Simultaneously, the health system should ensure that diabetes patients living in rural areas and belonging to ethnic minorities can access better healthcare services to improve their health and decrease their risk for chronic disease and death.

## Introduction

**Figure 1:**
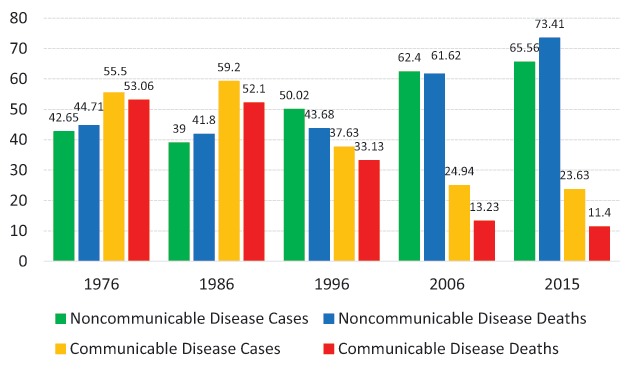
Morbidity and Mortality Trends in Vietnam during 1976–2015 (Unit: %) 6–8.

The prevalence of noncommunicable diseases (NCDs) has increased in low- and middle-income countries. It is worth noting that almost *three-quarters* of the population *aged 30–70 years has diabetes* [1]. Economic growth and changes in consumption and living styles coupled with changes in the social environment have influenced *disease patterns* ranging from communicable diseases (CDs) to noncommunicable diseases. According to global diabetes statistics, approximately 5% of those aged 35–39 years, 10% of those aged 45–49 years, 15% of those aged 55–59 years, and approximately 20% of those aged 65–69 years have diabetes [2]. Population aging is also increasing dramatically throughout the world, especially in developing countries. In Vietnam, such as in other countries, the population of those over 60 years old has created pressures on the health system as well as social security services and policies. Thus, Vietnam faces the problem of “getting old before getting rich” [3]. In Vietnam, health statistics reveal that noncommunicable disease deaths have increased from 44.07% in 1976 to 73.41% in 2015 (Figure 1). In contrast, communicable disease death decreases from 53.06% to 11.4% during the same period (Figure 1). Diabetes is a leading cause of death worldwide, and it causes a 30% loss of life expectancy [2]. In Vietnam, diabetes is projected to be one of the top seven diseases leading to death and disability in Vietnam by 2030 [4,5].

The burdens of double diseases (CD and NCDs) and the transformation of disease patterns put forward challenges for long-term health services for the elderly population and thus lead to an economic burden on family and society. Vietnam’s population has reached over 96.2 million in 2019, which means it is ranked 15th in the world and third in Southeast Asia [9]. The elderly population (aged 60 or above) of the total population reached 10% in 2017 [6]. Approximately 65.6% of the Vietnamese population lives in rural areas with lower living standards than those in other nations in Southeast Asia; they are only higher than those of East Timor (31%), Myanmar (29%) and Cambodia (23%) [3,9]. Followers of the Kinh (Viet) religion account for 87% of the population, and they live mainly in the significant delta areas and coastal plain. The followers of other religions (53 ethnic minority *groups*) mostly reside in mountainous areas and highlands [10]. The estimates of the prevalence of type 2 diabetes (T2D) were 2.7%, 5.4%, and 6% in 2002, 2012 and 2017; studies unveiled that the proportion of people with diabetes was increased compared to the finding of previous studies from the 1990s (only 1.2%) [2,4,11,12,13]. Furthermore, the Vietnam Burden of Disease and Injury Investigation showed that chronic diseases were the leading causes of death, accounting for 66% of all deaths in 2009, which increased to 78% in 2015 [14]. The fourth leading cause of death in women is diabetes [14], and diabetes-related deaths doubled from 2009 to 2015 (Table 1). Moreover, the risk of premature death between the ages of 30–70 years *from NCDs* (cancers, diabetes, cardiovascular diseases, and chronic respiratory diseases) *was* 17% in 2016 [15]. Many studies have reported that in 40–73% of cases, people are unaware that they have diabetes mellitus (DM) [16,17]. The proportion of patients with undetected diabetes in the community was still very high in 2012, at 63.6% compared to 64% in 2002 [18]. It has also been reported that the prevalence of diabetes in urban areas is 1.68 times higher than that in rural areas [17]. According to the national survey from 2002–2003, the rate of pre-diabetes was 9.2% in the country and 9.3% in the mountain area [18]. Diabetes is related to the influence of pathogenic factors such as cultural characteristics, living conditions, diet, and physical activity of each ethnic group [17,19,20,21]. However, there have been no studies conducted in the provinces with ethnic minorities to analyze the incidence of diabetes among these ethnic groups [22]. A cross-sectional study identifying the perception of and factors associated with DM showed that participants aged 60–70 years were more likely to have DM than those aged 30–39 years, and participants classified as obese were more likely to have DM than those with a healthy or low BMI [23]. The results of a multivariate logistic regression model unveiled the proportion of the Kinh ethnic group with diabetes as being 19.7 higher than that of an ethnic minority group, and this difference was statistically significant (OR = 19.7; 95% CI: 1.4–283.9) [24]. Given the prevalence of diabetes among different groups of people, especially vulnerable subjects such as inhabitants of the mountainous and remote areas, early detection is significant for the prevention and control of diabetes.

**Table 1:** Diabetes case and diabetes-related deaths from 2009 to 2015 [39,58,59,60,61].

Variables/years		2009	2011	2013	2015

1.	Mean diabetes-related expenditure per person (20–79 years old) with diabetes (USD)	62	123	128	163
2.	Diabetes cases (20–79 years old) in 1000s	1,643	1,702	3,299	3,509
3.	Diabetes-related deaths	32,505	27,949	54,953	53,457
4.	Adult population (20–79 years) in 1000s	56,661	58,408	61,387	61,697

## Changes in Diabetes Prevention and Risk Factors

There is a combination of genetic, physiological, environmental, and behavioral factors that encourages diabetes risk factors, including smoking, irrational nutrition, insufficient physical activity, obesity, hypertension, hyperglycemia [18].

### Biological/Metabolic risk factors: Obesity, Hypertension, Dyslipidemia, Hyperglycemia

**Figure 2:**
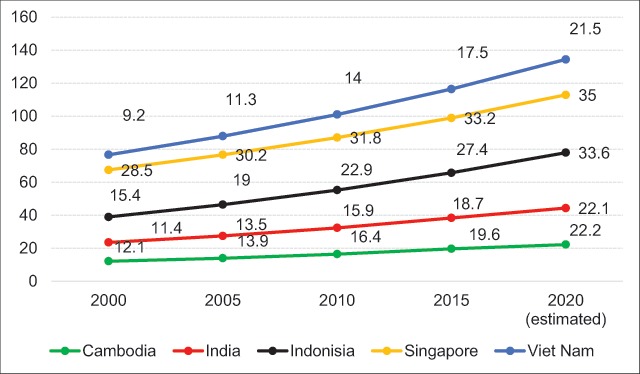
The trend of overweight and obesity in those aged 18 years and older from 2000–2020 (Unit: %) 31.

In recent years, obesity has become one of the most influential contributors to T2D. Approximately 20% of people who die of cancer are overweight or have high BMI (BMI ≥ 25 kg/m^2^) [25]. The WHO estimated that 39% of adults aged 18 years and older were overweight in 2016, and 13% were obese [26]. According to STEPS, the prevalence of overweight among Vietnamese adults in 2015 (15.6%) was almost five times that of 2000 (3.5%); the BMI among urban populations is significantly higher than that among rural populations (21.3% vs. 12.6%, respectively) [20,27,28]. The prevalence of overweight/obesity (combined) is similar in males (33.6%) and females (31.5%) and generally increases with age, especially for those 50–60 years of age, reaching 36.9% [29]. Assessing the overweight and obesity rates of adults aged 25–74 years (using Asian-specific BMI cut-offs) shows that the prevalence of overweight and obesity are 28.6% and 2.1%, respectively [30]. The prevalence of overweight and obesity among the mountain population is 28.2% and 43.8%, respectively [21]. Based on the data, we estimated that the BMI rate increased 1.9 times during 2000–2015 [31]. It is estimated that if the increase in BMI continues to follow a similar trend, then the proportion of adults being overweight will be 21.5% by 2020 (estimated) (Figure 2). Vietnam’s obesity rate seems to have increased doubly compared to that of other countries in the period 2000–2015; Cambodia and India experienced an increase of 1.6 times, Indonesia’s increase was 1.77 times, and Singapore’s increase was 1.1 times (Figure 2).

The prevalence of hypertension reported in STEPs in 2015 was significantly higher than that reported in STEPs in 2010, increasing from 15.3% (95% CI: 14.9–15.7) to 20.3% (95% CI: 18.5–22.1) among the 25–64 age group [8,20,32]. The prevalence of high blood cholesterol (≥5 mmol/L) remained high (30.2%), in 2015, with no change from the prevalence in 2010 [18,20]. Thus, the proportion of adults with raised blood cholesterol would increase the proportion of cases at risk of NCD, particularly diabetes. Additionally, approximately 56.9% of people with hypertension and 68.9% of people with diabetes were undiagnosed [20]. Obesity and hypertension are of concern due to the related increase in healthcare costs. It creates a considerable burden on both families and society to pay the health care costs related to the prevention and treatment of diabetes and other diseases.

### Risk behaviors: Smoking, Irrational nutrition, Insufficient physical activity

#### Smoking and Lack of knowledge about diabetes

The WHO estimates that in 2015, 20.2% of those aged ≥15 years for both sexes were current smokers (367 million), while the prevalence of those who smoked in Southeast Asia was 27.7% (191.8 million) [33]. Vietnam is one of 15 countries with the highest number of tobacco users in the world [18]. For both sexes, approximately 23.8% of the population were tobacco smokers in 2010, which slightly decreased to 22.5% in 2015 [18]. Each year, tobacco use causes more than 40,000 deaths in Vietnam, which accounts for more than 100 deaths/day; this number will increase to 70,000 death per year by 2030 [34]. In recent years, the diabetes prevention program at the Centers for Disease Control and Prevention (CDC) has facilitated primary health care, which includes suggestions on how to empower patients, help them to take control of their blood sugar and follow a self-controlled diet [35]. However, the findings suggest that 67% of the participants had never heard of DM [23], and only 3.9% had a moderate or above knowledge level about the dangers and complications of the disease; additionally, only 0.6% had experience with the risk factors, and 21.9% had knowledge about the prevention and treatment of diabetes [18]. Abdominal obesity (obesity by the waist-hip ratio [WHR]) was positively associated with female gender (OR 43.64, 95% CI 13.15–144.86); however, it was negatively associated with smokers and people aged ≥60 in ethnic groups other than the Kinh and Tay religions [21]. More than 88% of the inhabitants disagreed with the statement that the treatment of T2D and its complications is not necessary [36]. This result shows that knowledge about diabetes and attitudes towards the condition are known to affect compliance and play an essential role in diabetes management. In general, the experience of T2D treatment was significantly lower in rural areas than in urban areas [36]. In practice, some studies have shown that the proportion of patients adhering to diabetes treatment is low at 14.2% [37]. In 2010, diabetes accounted for 3% of the deaths in older adults aged approximately 70 years in Vietnam [38].

#### Irrational nutrition

Vietnam has the double burden of over- and undernutrition [28]. Concerning NCDs, 80.6% of people do not consume the recommended number of five servings of fruit and vegetables, and they have diets that are high in salt, fat, and sugar [39]. Studies have found that 31.3% of the total deaths and 25.3% of the whole disability-adjusted life years (DALY) in Vietnam were caused by an unhealthy diet [40]. The DALY combines the estimates of years of life lost due to premature death (YLL) and years lived in ill health or with disability (YLD) to count the total years of functional experience lost from diseases [40]. Researchers have concluded that the leading risk factor for diabetes-related diseases is lifestyle and dietary issues [41].

#### Physical Inactivity

Studies have shown that physical activity could help to prevent heart disease and diabetes, improve sleep, and lower the risk of falls, obesity, and high blood pressure in the aging population [42]. Also, physical activity has *many* benefits related to controlling blood glucose and decreasing risk factors for mortality. However, approximately 3.2 million deaths and 32.1 million DALYs (representing approximately 2.1% of the global DALYs) are attributable to insufficient physical activity [38,43]. The results from the 2010 STEPS survey in Vietnam showed that the percentage of adults who got inadequate physical activity was 28.7% (26.4% for males, 30.8% for females) and that the rate of insufficient physical activity in urban areas was higher than that in rural areas, at 36.9% and 25.1%, respectively [44]. Thus, it is imperative that more coordinated educational campaigns and programs are implemented, with a prioritized focus on weaker, rural, and less-educated groups [45]. From 2009–2010, more than a quarter of the adult population was insufficiently active, with a more significant proportion of inactive females than males in the 25–64 age group [32].

**Figure 3:**
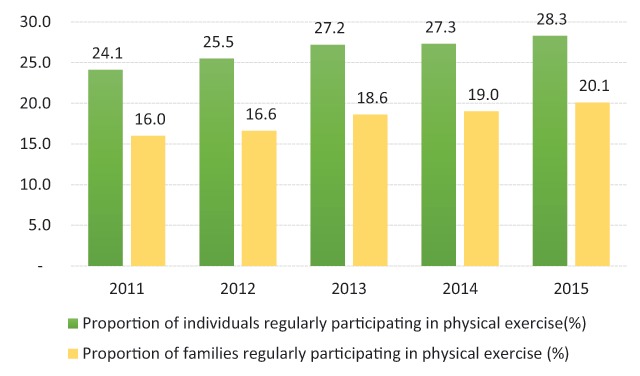
Proportion of individuals/family periodically participating in the physical activity [46].

**Figure 4:**
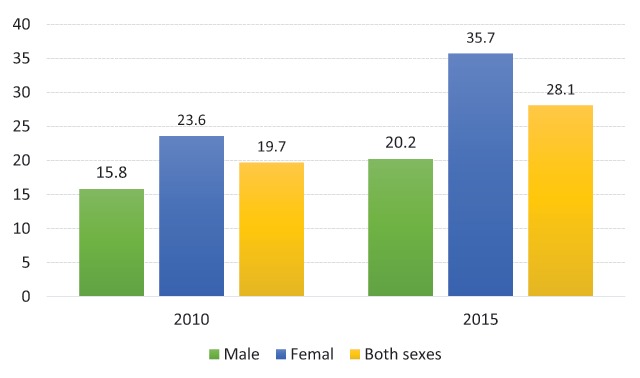
Prevalence of low physical activity, adults aged 25–64 years (Unit: %) [18,20,32,47,48,49].

Only a few studies have measured the proportion of individuals and families that participated in regular physical exercise from 2010–2015 (Figure 3) [46]. According to the results of the second national Survey and Assessment of Vietnamese Youth (SAVY) in 2009, up to 23% of youth rarely or never performed physical exercises or sports, and 45% “sometimes” did so [47]. There are considerable differences between the prevalence of families and individuals regarding physical activity (Figure 3). More than a quarter of the adult population aged 25–64 years are insufficiently active, with a higher proportion of inaction in females than in males (Figure 4). Figure 4 shows that the prevalence of low physical activity for both sexes was 28.1% in 2015. This figure was lower among males (20.2%) compared to females (35.7%) in regards of not meeting WHO recommendations on physical activity for health. Thus, effective lifestyle modifications, including counseling on weight loss and the adoption of a healthy dietary pattern, together with physical activity, are the cornerstones of the prevention of T2D [19]. There is a lack of enabling environments and convenient physical facilities to support people in enhancing their physical exercises to improve their health, especially in schools at different levels, in offices, in workplaces and in public places [18].

### Access to services for the prevention, diagnosis, and treatment of diabetes is difficult

Health service delivery (HSD) is a part of the health system in which patients receive the necessary treatment and supplies [6]. In general, primary health care practitioners in low-income countries do not possess the necessary technologies needed to help people with diabetes properly manage their disease. Access to essential medicines, including life-saving insulin and techniques, are limited in low- and middle-income countries [50]. When the participation of all levels, sectors, and communities in the first phase is limited, even within families, the patients do not have the full awareness of the symptoms of the disease needed to take actions, and this is especially true for older adults [18]. When the management of the diabetes is left to the sick, it creates more of a burden for the family and affects the development of society [3].

HSD includes both *public and private healthcare* providers in Vietnam (60% of outpatient service providers are at the private level) [8,51]. The communes are all located in mountainous or remote areas and are approximately 10 to 40 km away from a district health center [52]. While 71.6% of the population held public health insurance in Vietnam by the end of 2014, many of the primary care facilities do not possess the capacity to diagnose, treat and monitor diabetes patients, or provide follow-up care for patients [18].

The target for the period 2016–2020 is to reach a rate of 40% CHS involved in the treatment and management of hypertension, diabetes, and some other noncontagious diseases [53]. Using the fundamental technologies available in primary care facilities, blood glucose measurement, oral glucose tolerance tests, HbA1c tests, foot vibration perception by tuning fork, and urine strips for glucose and ketone measurements are not available [54]. Therefore, there is a need to significantly influence the access to and use of health services in primary health care settings. Data from the National Health Survey in 2001–2002 showed that the proportion of illnesses treated by self-medication in the whole country accounted for 73% [55]. Meanwhile, the ability of the health system to respond is limited, especially regarding the medical examination, treatment, disease control and management of NCDs in the community, which leads to overloaded hospitals and an increase in substance use. Currently, CHSs have not met the needs of the patients. In addition to the above reasons, self-treatment is a relatively common behavior in Vietnam [18].

## Health Expenditure

Differences in geographical, economic, and social conditions between the delta and mountain areas and between rural and urban areas, also affect the health status of people with diabetes [16,17,21]. The growing trend of diabetes has caused an increasing demand for medical treatment and treatment services at all health facilities. The cost of treating diabetes is complex and involves multiple factors that are higher than basic health costs, which are not affordable to poor or near-poor individuals, especially people in rural and mountain areas [56]. In particular, T2D is associated with an increase in the rate of chronic complications, which causes a great deal of damage to the human body and an individual’s physical strength and also affects the economic development process of each country [1]. Thus, the burden of disease associated with diabetes is substantial; in financial terms, direct health care costs continue to increase and are currently at 12% of the global health expenditure, while indirect damages such as the loss of production may be five times this number [39,50]. Studies in health economics have also shows that even in prediabetes, patients have a high risk of developing T2D, which increases the cost of health care [56]. In Vietnam, the health expenditure per capita is $122.84 [57]. According to the International Diabetes Federation, the mean diabetes-related spending per person (aged 20–79 years) with diabetes increased from $62 in 2009 to $163 in 2015 (Table 1) and $217 in 2017 [2], and there are approximately more than 5 million people with diabetes in the community, 3.5 million of whom are adults (aged 20–79 years) with diabetes (Table 1) [39]. People with diabetes are likely to seek more medical health care and accrue more health care costs than are those without the risk of developing diabetes.

Although current health expenditures tends to be lower than those in the past due to the policy of supporting health insurance for poor and near-poor individuals, the poverty rate is likely to increase in these two groups, with a 6% rate for the near-poor population and 5.4% for the poor population, while the rate was only 0.1% for the rich and affluent during 2010 [62]. Additionally, the cost of outpatient treatment is much higher than that of inpatient treatment. On average, a poor household spends 47.12% of their total income on diabetes, with the highest spending being related to medications [63]. Diabetes is a chronic disease that requires regular treatment, daily medication, hospitalization, or other complications [63]. Therefore, the cost of diabetes is an economic burden on older people and their families in Vietnam. Although health insurance has significantly shared the household costs for diabetes-related diseases, health insurance is also a barrier for people who participate in voluntary insurance.

## Health Systems and Healthcare Polices in Vietnam

**Figure 5:**
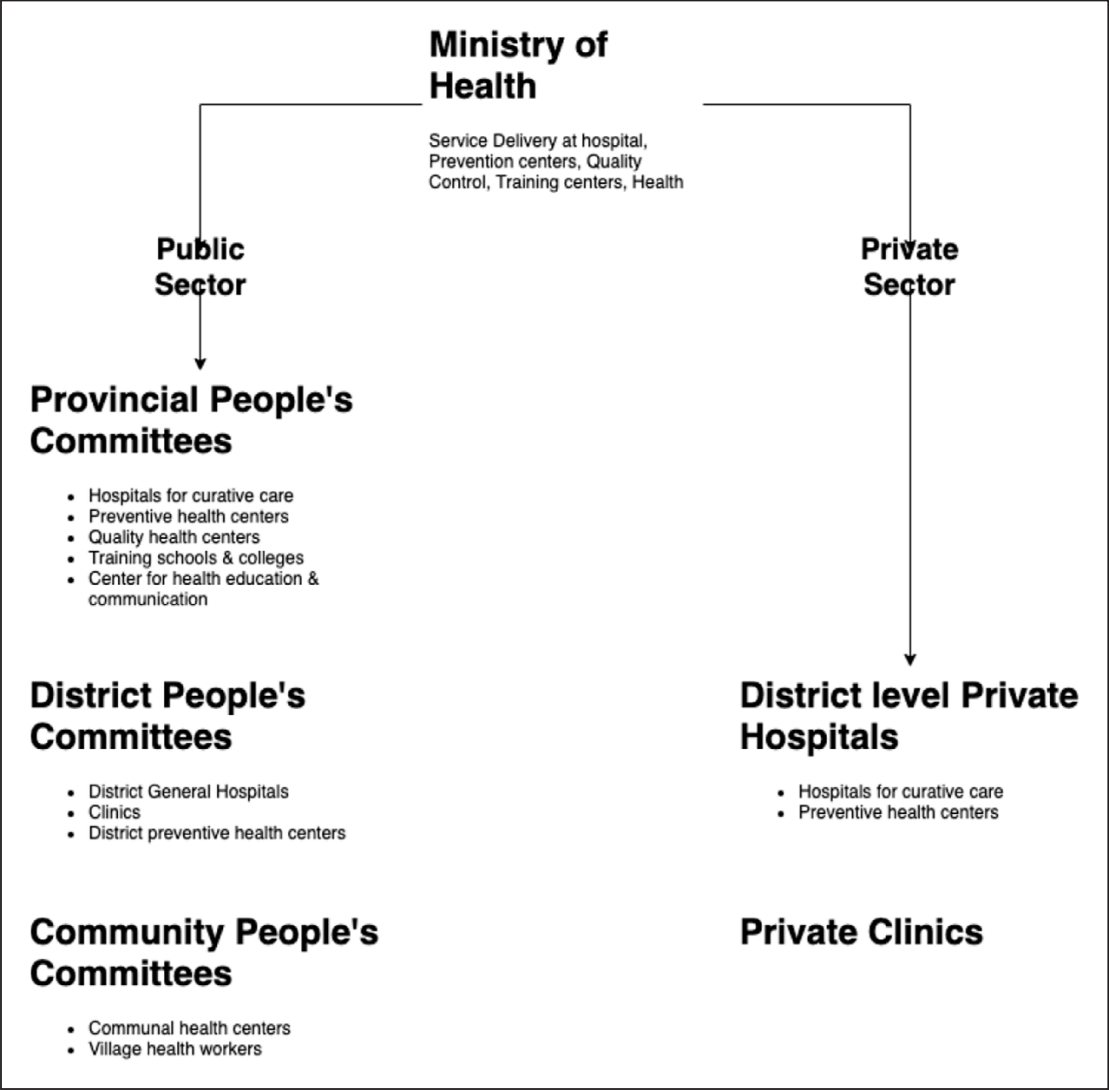
Healthcare delivery in Vietnam.

The treatment and preventive medicine systems in Vietnam are multilevel under the Ministry of Health (MOH). The health system in Vietnam consists of four levels of service: central, provincial, district, and commune health stations (CHSs) (Figure 5) [18,20]. Additionally, there are national institutes, such as the National Institute of Nutrition and the National Institute of Hygiene and Epidemiology [27]. There are approximately 1,488 hospitals in Vietnam, with 47 at the central level, 459 at the provincial level and 982 district hospitals; CHSs are responsible for providing 99% of the primary health care services for people in the commune (Table 2), and only 66% of these CHSs have a medical doctor [64].

**Table 2:** Number of facilities providing health services [6,7,8,48].

Facilities/Year	2008	2012	2017

	Number	Number	Number

Central Level	21	46	47
Provincial Level	64	434	459
District Level	622	1,310	982
Communal level	10,396	11,105	11,120

According to Decision No. 376/QĐ-TTG of the Prime Minister, there is a need to set a target for strengthening the prevention, management and treatment of several NCDs at CHSs, especially for the management of hypertension and diabetes, in an attempt to provide better primary care at local levels and to reduce the burden on central and provincial medical facilities [64]. However, public hospitals and clinics in Vietnam are frequently underfunded and poorly equipped. In addition, the proportion of older adults continues to increase, leading to the increased demand for long-term health care services [65]. Vietnam bears the double burden of disease, the aging population is rapidly increasing, and annual physical exams are *not fully* covered. So the goal of the Vietnamese health financing system towards universal health insurance is an aim of fairness, quality, and efficiency [64]. The percentage of the GDP spent on health changed significantly from 4.85% in 2000 to 5.66% in 2016 [66]. Compared to many countries in the region, such as Malaysia (3.8% of GDP in 2016), the Philippines (3.5% of GDP in 2016), and Singapore (4.47% of GDP in 2016), Vietnam’s healthcare costs are relatively high [66]. The government has launched Decree 146/2018/NĐ-CP (2018) to create the best conditions for patients with chronic diseases, people with disabilities, and older persons as well as to help them seek *medical services* and save *the costs* of *medical care* and time [67]. As a result, patterns of health expenditures and financial protections in Vietnam from 1992–2012 show that health insurance coverage increased from 10% in 1995 to 68.5% in 2012 [68]. The government is working on improving the health insurance coverage, which reached 77% of the population in 2015 [6,8,18]. It is also focused on enhancing the capabilities of the healthcare infrastructure and wellness rather than sickness, especially in terms of the conditions in rural areas. However, some issues negatively impact access to services, including poor equipment and the lack of essential treatment services in CHSs, which compromise the ability of governments to provide healthcare to citizens [18]. The Vietnamese government is making efforts to improve public healthcare in rural areas through Project 1816 (2017), which is also known as the satellite hospital project [18]. The aim of the satellite hospital project is that it motivates provincial-level hospitals to upgrade their infrastructure and invest in training medical staff, which in turn brings benefits to patients.

## Conclusions and Strategies for Prevention of Diabetes in Vietnam

The results of this study provide a discussion on subjects, including high-risk factors for diabetes. Control of the principal risk factors, including smoking, unhealthy diets, physical inactivity, and other factors, along with active surveillance, early detection, treatment, long-term and continuing management at primary health care facilities, are key measures. Therefore, there is a need to develop and implement policies on risk factor prevention, such as tobacco control policies that reduce the demand for tobacco and the management of the tobacco supply [18,34]. Policies that encourage the provision of services for the prevention, treatment, and control of diabetes in the community through family and village doctors, especially in mountain and rural areas, are also needed. In addition, essential medicines and testing equipment are needed to offer sufficient treatment of diabetes at CHSs. Thus, strengthening the system of examination and treatment facilities to provide comprehensive, advanced, and high-tech services in the diagnosis and treatment of patients with diabetes at the CHSs and district level is also needed. Vietnam is currently striving towards a universal healthcare system to decrease healthcare costs. However, the insurance system should ensure long-term and continuing care and treatment for older individuals with diabetes in the mountain areas. Simultaneously, the health system should ensure that diabetes patients living in rural areas and belonging to ethnic minorities can access better healthcare services to improve their health and decrease their risk for chronic disease and death. Therefore, future studies need to consider access to healthcare and the quality of life of people with diabetes and promote the role of families in the management, care, prevention, and education related to diabetes.
